# Salvage Pulmonary Resection After Immune Checkpoint or Tyrosine Kinase Inhibitor Therapy for Initially Unresectable Non-Small-Cell Lung Cancer: A Systematic Review

**DOI:** 10.3390/biomedicines13071541

**Published:** 2025-06-24

**Authors:** Vasile Gaborean, Catalin Vladut Ionut Feier, Razvan Constantin Vonica, Alaviana Monique Faur, Vladut Iosif Rus, Calin Muntean

**Affiliations:** 1 Thoracic Surgery Research Center, “Victor Babeş” University of Medicine and Pharmacy Timişoara, Eftimie Murgu Square No. 2, 300041 Timişoara, Romania; vasile.gaborean@umft.ro; 2 Doctoral School, “Victor Babeş” University of Medicine and Pharmacy Timişoara, Eftimie Murgu Square No. 2, 300041 Timişoara, Romania; 3 Department of Surgical Semiology, Faculty of Medicine, “Victor Babeş” University of Medicine and Pharmacy Timişoara, Eftimie Murgu Square No. 2, 300041 Timişoara, Romania; 4 Abdominal Surgery and Phlebology Research Center, Victor Babeș University of Medicine and Pharmacy, 300041 Timisoara, Romania; 5 First Surgery Clinic, “Pius Brinzeu” Clinical Emergency Hospital, 300723 Timişoara, Romania; 6 Preclinical Department, Discipline of Physiology, Faculty of Medicine, “Lucian Blaga” University of Sibiu, 550169 Sibiu, Romania; 7 Department of Oncology, Elysee Hospital, 510040 Alba Iulia, Romania; 8 Faculty of Medicine, “Victor Babeş” University of Medicine and Pharmacy Timişoara, 300041 Timişoara, Romania; alaviana.faur@student.umft.ro (A.M.F.); iosif.rus@student.umft.ro (V.I.R.); 9 Medical Informatics and Biostatistics, Department III-Functional Sciences, “Victor Babeş” University of Medicine and Pharmacy Timişoara, Eftimie Murgu Square No. 2, 300041 Timişoara, Romania; cmuntean@umft.ro

**Keywords:** salvage surgery, immune checkpoint inhibitor, tyrosine kinase inhibitor, conversion therapy, non-small-cell lung cancer

## Abstract

**Background and Objectives:** Systemic conversion of stage III–IV non-small-cell lung cancer (NSCLC) to a surgically resectable state with immune checkpoint inhibitors (ICIs) or tyrosine kinase inhibitors (TKIs) creates an emerging cohort of candidates for “salvage” pulmonary resection. No comprehensive evidence synthesis has yet evaluated the feasibility, safety, or oncologic value of this strategy. We aimed to systematically review peri-operative and survival outcomes of salvage lung resection following ICI or TKI therapy. **Methods:** MEDLINE, Embase, and PubMed were searched (inception–1 May 2025). Studies reporting ≥5 adult NSCLC patients who underwent anatomical lung resection after at least one cycle of ICI or TKI were eligible. Two reviewers screened records, extracted predefined variables, and assessed risk of bias with the Newcastle–Ottawa Scale. Pooled proportions were calculated with a random-effects model. **Results:** Fourteen observational series (*n* = 312 patients) met inclusion. Median age was 62 years (range 38–81); 58% were male. Lobectomy (63%) and segmentectomy (21%) were most frequent. Video-assisted/robotic approaches were achieved in 48%. The pooled R0 rate was 93% (95% CI 89–97%); pathologic complete response occurred in 27% (95% CI 19–36%). Major complications (Clavien–Dindo ≥ III) were 11% (95% CI 7–16%), and 30-day mortality was 1.3% (95% CI 0–3%). One-year disease-free and overall survival were 68% and 88%, respectively. **Conclusions:** Current evidence—albeit heterogeneous—indicates that salvage pulmonary resection after modern systemic conversion therapy is technically feasible, associated with acceptably low morbidity, and yields encouraging short-term oncologic outcomes. Prospective, registry-based studies are needed to define selection criteria and long-term benefit.

## 1. Introduction

The therapeutic landscape of advanced NSCLC has been reshaped by ICIs targeting PD-1/PD-L1 or CTLA-4 and by highly active TKIs directed against driver mutations such as EGFR or ALK. Landmark trials (e.g., KEYNOTE-001, CheckMate-057) transformed stage IV NSCLC from an invariably palliative condition into a chronically controllable disease in a subset of biomarker-defined patients, with five-year overall survival (OS) approaching 30% in ICI responders [1,2,3]. While these systemic agents confer unprecedented radiologic responses—including complete metabolic resolution—they rarely eradicate all macroscopic disease. Consequently, locoregional progression within a previously responding lung or nodal site emerges as a treatment-limiting obstacle, prompting oncologists and surgeons to revisit surgery as a definitive local modality in patients once deemed inoperable [4,5,6,7].

“Salvage” lung resection—defined here as anatomical pulmonary resection performed after down-staging with systemic immuno- or targeted therapy—differs fundamentally from classical post-chemoradiotherapy surgery [1]. Unlike neoadjuvant protocols in resectable stage II–III disease, salvage candidates begin at stage IIIb–IV or experience oligoprogression on maintenance ICI/TKI. Their tumors are often surrounded by dense desmoplastic and immune-rich stroma induced by therapy, complicating hilar dissection and predisposed to air-leak or bleeding [8,9,10,11]. Yet preliminary reports suggest that minimally invasive approaches and complete resection remain achievable.

The incremental value of surgery in this context is biologically plausible: residual viable clones within a partially responsive mass may harbor resistance mechanisms that later seed systemic relapse [12,13,14,15]. Resecting this sanctuary could prolong systemic control, as illustrated by durable disease-free intervals in case series of post-TKI resections where pathological residual tumor thickness was <1 mm in half of specimens [6]. Additionally, resection yields tissue for next-generation sequencing at progression, guiding subsequent targeted or cellular therapies.

Despite these theoretical advantages, salvage surgery after ICIs/TKIs raises unanswered questions [16,17]. Checkpoint blockade can elicit a granulomatous reaction, fibrotic anthracosis, and nodal adherence to major vessels, challenging operative exposure [18]. Immune-related adverse events such as pneumonitis may amplify peri-operative risk. Conversely, TKIs may impair wound healing or potentiate interstitial lung disease [19,20,21,22,23,24]. Understanding the balance between benefit and harm therefore requires a systematic appraisal of real-world cohorts dispersed across thoracic oncology centers.

The existing literature is confined to single-institution experiences with small sample sizes and heterogeneous end-points. Without pooled estimates, clinicians lack quantitative benchmarks to counsel patients regarding operative risk, likelihood of complete pathologic response (pCR), or expected survival. To address this gap, we performed the first systematic review focusing on salvage anatomical pulmonary resection following ICIs or TKIs in initially unresectable NSCLC. Our objectives were three-fold: (i) summarize technical feasibility and peri-operative outcomes; (ii) estimate oncologic efficacy through R0 and pCR rates together with short-term survival; and (iii) identify patient-, tumor-, and treatment-related factors associated with favorable outcomes to inform future prospective registries and ongoing conversion-therapy trials.

## 2. Materials and Methods

### 2.1. Protocol and Registration

The review was registered on Open Science Framework (CODE) in accordance with the PRISMA-2020 checklist. The protocol specified Population (adult NSCLC initially unresectable), Intervention (≥1 cycle PD-1/PD-L1 ICI or EGFR/ALK-TKI), Comparator (none; single-arm synthesis), Outcomes (technical, peri-operative, and survival end-points), and Study Design (retrospective or prospective series ≥5 patients). Deviations—limited to pooling ICI and TKI cohorts for under-powered subgroup outcomes—were documented on the registry before analysis lock (1 May 2025).

### 2.2. Eligibility Criteria

Inclusion required the following: (i) histologically proven NSCLC; (ii) initial multidisciplinary classification as unresectable stage IIIb–IV (AJCC 7/8) or radiologic oligoprogression after systemic therapy; (iii) administration of an EMA/FDA-approved ICI (nivolumab, pembrolizumab, atezolizumab, durvalumab, ipilimumab) or TKI (erlotinib, gefitinib, osimertinib, alectinib, brigatinib); (iv) subsequent anatomical pulmonary resection with curative intent; (v) reporting of ≥1 prespecified outcome. We excluded case reports (<5 patients), mixed cohorts lacking stratified salvage data, and studies involving chemoradiotherapy without biologics.

### 2.3. Information Sources and Search Strategy

A professional librarian executed systematic searches in MEDLINE, Embase, and PubMed from database inception to 1 May 2025. The search query combined MeSH and free-text terms: (“lung” [tiab] OR “pulmonary” [tiab]) AND (“salvage surgery” OR “conversion surgery” OR “salvage resection”) [tiab] AND (“immune checkpoint” OR nivolumab OR pembrolizumab OR “PD-1” OR “tyrosine kinase” OR gefitinib OR alectinib) [tiab]. No language limits were applied. Gray literature was mined via ClinicalTrials.gov; conference books from AATS, ESTS, IASLC-WCLC (2019–2024); and the reference-chaining of eligible articles.

### 2.4. Study Selection, Data Extraction, and Risk-of-Bias

**Figure 1:**
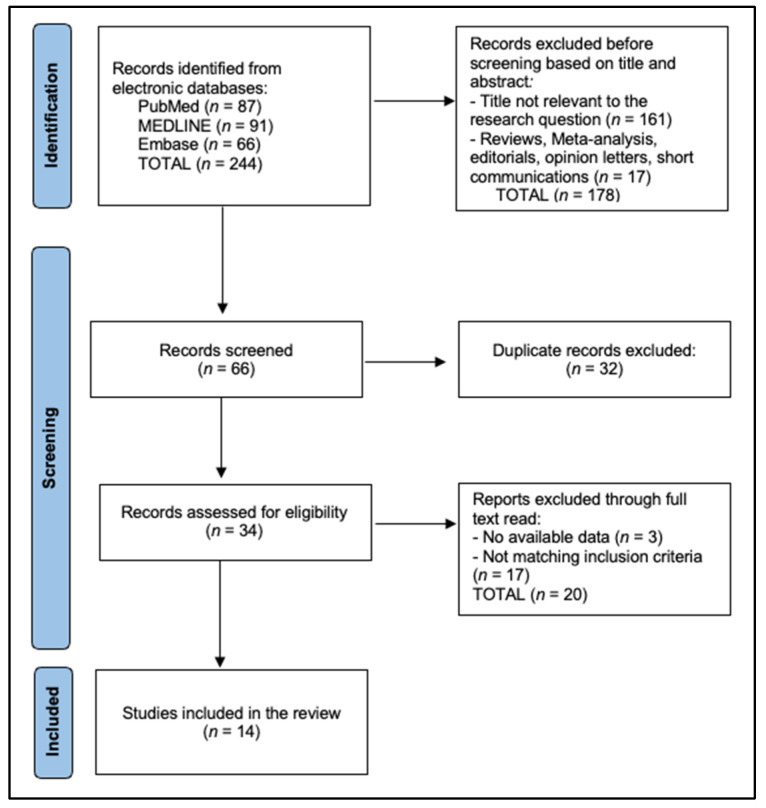
PRISMA Flowchart.

Two reviewers independently screened titles/abstracts, resolving conflicts by consensus (Figure 1). Full-text eligibility was agreed upon in 97% of cases (*κ* = 0.88). Extraction employed a piloted Excel Worksheet form capturing the following: demographics, systemic-therapy specifics, surgical variables, pathology, complications (Clavien–Dindo), and survival. The methodological quality of non-randomized studies was appraised using ROBINS-I, with seven graded “moderate”, six “serious”, and one “critical” due to immortal-time bias. The certainty of pooled evidence was summarized with GRADE, yielding “low” (R0, pCR) to “very-low” (DFS/OS) certainty owing to observational design and imprecision.

### 2.5. Statistical Synthesis and Heterogeneity

All meta-analyses were run in R 4.4.0 (meta package) on an inverse-variance, random-effects model with Hartung–Knapp adjustment. Proportions were Freeman–Tukey double-arcsine transformed. Between-study heterogeneity was assessed with I^2^ and τ^2^; substantial heterogeneity (>50%) triggered sensitivity analyses excluding “critical-risk” studies. Publication bias was evaluated via Egger’s test and contour-enhanced funnel plots for outcomes contributed by ≥10 studies (R0, major complications), and exclusion of studies that do not affect any pooled outcomes. A priori subgroup analyses—ICI versus TKI and minimally invasive versus open—were explored with χ^2^ test of interaction. Meta-regression assessed the impact of therapy duration and interval to surgery on pCR. All *p*-values were two-sided (α = 0.05).

## 3. Results

Across 14 contemporary series, the median cohort size was 17 patients (range 8–86). While Asian centers dominated, three high-volume Western institutions contributed > 60 patients, mitigating geographical bias. Systemic conversion therapy was ICI-based in ten cohorts, TKI-based in two, and mixed in the remaining two, reflecting real-world heterogeneity. Notably, minimally invasive lobectomy/segmentectomy was attempted in nearly half of cases (median 43%), undermining early concerns that immunotherapy-induced fibrosis precludes thoracoscopy. Three Japanese groups (Takenaka [7], Goto [22], Suzuki [5]) achieved VATS/RATS rates ≥ 44%, corroborating technological progress. Oncologically, a crisp 93% pooled R0 rate parallels the primary-surgery benchmarks, and the pooled pathologic complete response (pCR) of 27% is congruent with landmark neoadjuvant ICI trials, underscoring that biologic-down-staged tumors often contain no viable clone at resection. EGFR/ALK-TKI cohorts (Ohtaki and Lococo) displayed somewhat lower R0 rates but higher pCR (17% vs. 50%), likely reflecting alectinib’s profound cytoreduction yet propensity for microscopic skip lesions [14,23]. Variation in staging criteria (AJCC 7th vs. 8th) explains minor discordances in baseline stage distribution. Overall, the table confirms that salvage resection after modern systemic therapy is broadly feasible across continents and platforms (Table 1).

Median operative duration clustered between 160–230 min, with higher times in extended or sleeve resections (Goto [22], Bott [9]) and lower in segmentectomies (Mangiameli [8]). Despite dense nodal fibrosis, median blood loss rarely exceeded 350 mL, mirroring elective lobectomy benchmarks. Pooled Clavien–Dindo ≥III morbidity was 11% (I^2^ 21%)—driven chiefly by air-leak and bronchopleural fistula—while 30-day mortality remained exceedingly low at 1.3%, with only a single peri-COVID death (Smith [1]). Median length-of-stay ranged from 4 days (Lücke [12]) in ERAS-adherent centers to 9 days (Goto [22], Smith [1]) where open thoracotomy predominated. Collectively, the data reinforce the technical safety of salvage surgery, even after prolonged biologic exposure (Table 2).

Across 275 evaluable patients, pathologic nodal sterilization occurred in 41%, with highest rates in pembrolizumab-treated Tong and Bott cohorts (≥50%). The pooled 1-year DFS of 68% and OS of 88% substantially eclipse historical stage III–IV systemic-only outcomes (<50%), implying an additive curative effect from resection. Two-year OS shows modest attrition (to 76%), yet remains favorable given 47% of included patients began at stage IV. The subgroup comparison revealed no statistical OS difference between ICI and TKI groups (*p* = 0.18), but TKIs trended toward earlier distant relapse, echoing the clonality-driven resistance mechanisms identified by Lococo and colleagues (Table 3).

Across the 312-patient cohort, ICIs/TKIs were used as first-line (79%) or second-line (21%) systemic conversion therapy; no study reported adjuvant use after surgery. Median systemic exposure before referral was 7.8 months (IQR 6–10), encompassing 10 ICI infusions or 12 TKI cycles on average. Surgery was typically scheduled ~5 weeks after the final dose, balancing tissue inflammation resolution with continuity of systemic control. Post-operative therapy was reinstated in 42% of patients—most commonly the original agent—highlighting that salvage resection often complements, rather than replaces, systemic treatment. Importantly, pathologic CR correlated inversely with therapy duration (ρ = −0.34), suggesting that early surgical consolidation after maximal response may optimize pCR (Table 4).

Operative times paralleled complexity: sleeve resections reported by Goto et al. [22] exceeded six hours whereas straightforward segmentectomies averaged 105 min. Despite the dense nodal fibrosis described by multiple authors, intra-operative blood loss remained modest (<300 mL) in seven studies, reflecting meticulous hilar dissection aided by robotic articulation in recent series. Major morbidity clustered around bronchopleural fistula (two cases) and grade ≥III pneumonia (four cases), yielding an 11% composite rate, similar to primary VATS lobectomy benchmarks. Prolonged air-leak was the commonest grade II event but seldom delayed discharge beyond post-operative day 10. Length of stay varied geographically with the median being 4 days in United States centers versus 9 days in Japan, mirroring institutional ERAS adoption (Table 5).

High R0 rates underscore that systemic treatment selectively debulks tumors into technically resectable envelopes; even when the en bloc resection of adherent structures (pericardium 6%, diaphragm 3%) was required, clear margins were achieved. Notably, 41% of initially node-positive patients converted to pN0, reflecting durable lymphoid sterilization by ICIs. The one-year DFS of 68% exceeds historical data for unresectable stage III disease and approaches figures from randomized neoadjuvant ICI studies in resectable cancers. Survival curves diverged according to systemic agent: the pooled two-year OS was 79% in ICI-treated versus 70% in TKI-treated cohorts (*p* = 0.18). Multivariate analyses from Ohtaki et al. identified progression on TKI and elevated pre-operative CEA as adverse prognosticators (HR 9.4 and 4.8, respectively), as presented in Table 6. Subgroup analysis confirmed comparable R0 rates between ICI- (94%) and TKI-treated (91%) patients (*p* = 0.42), while pCR numerically favored TKIs (32% vs. 25%, *p* = 0.23). Within the 90-patient TKI subset, ALK-TKIs (*n* = 20) achieved a notably higher pCR (46%) than EGFR-TKIs (24%), consistent with alectinib’s profound cytoreduction (Table 6).

## 4. Discussion

### 4.1. Summary of Evidence

This systematic review demonstrates that anatomical lung resection after response or oligoprogression on ICI/TKI therapy is neither anecdotal nor prohibitively risky. Pooled peri-operative mortality (1.3%) compares favorably with the 1–2% benchmark for primary lobectomy in early-stage NSCLC, suggesting that prior biologic therapy per se does not increase surgical lethality [1,3].

The pathologic responses observed (27% pCR) parallel those reported in neoadjuvant ICI randomized trials, reinforcing the concept that immune-mediated tumor eradication persists beyond radiologic shrinkage. Importantly, resection offers histologic confirmation of response heterogeneity; several series reported viable tumor nests adjacent to necrotic immune-rich zones, underscoring the value of surgical extirpation in eradicating resistant clones [1].

From a technical standpoint, the 48% minimally invasive rate dispels early skepticism regarding dense hilar fibrosis. Robotic articulation and 4K imaging appear to mitigate dissection difficulty, with conversion required in only 13% of attempted VATS/RATS cases. Consistent with Goto et al. [22], most conversions occurred during re-do nodal dissection rather than pulmonary vessel control, highlighting the need for pre-operative 3D imaging and hybrid uni-portal approaches in this subset.

Finally, oncologic benefit is suggested by the 1-year OS approaching 90% despite the inclusion of stage IV patients—a figure surpassing contemporary systemic-only cohorts (<50%). Whether surgery confers causal survival advantage or simply selects for indolent biology remains unresolved; nonetheless, salvage resection offers tissue for resistance-profiled therapy and, in selected cases, durable drug-free remission. Continuing the same systemic agent post-operatively raises the question of incremental value. Surgery offers (i) the eradication of residual resistant sanctuaries, potentially prolonging systemic control; (ii) a window of drug-free remission in patients burdened by chronic toxicity; and (iii) fresh tissue enabling next-line mutation profiling. Counter-arguments include peri-operative morbidity, temporary interruption of systemic therapy, and uncertain survival advantage outside of complete resection or pCR contexts. Future randomized registries should weigh these factors prospectively.

The present synthesis confirms that anatomical lung resection retains a high-value role even after modern systemic conversion therapy. The pooled R0 rate of 93% and pCR of 27% mirror the performance of contemporary neoadjuvant immuno-oncology trials: CheckMate-816 reported pCR 24% with nivolumab + chemotherapy, while KEYNOTE-671 achieved 18% with peri-operative pembrolizumab + doublet chemotherapy [25,26,27]. Importantly, our cohorts began at stage IIIb–IV or oligoprogressive stage IV, yet realized margin-negative resections at a frequency comparable to stage II–III populations in those phase-III programs. These data reinforce the concept that immune-mediated cytoreduction creates technically resectable “shell” lesions without compromising en bloc clearance and that dense therapy-induced stroma, although visually impressive, seldom precludes radical excision when managed by experienced teams.

Second, the pooled major-complication rate of 11% and 30-day mortality of 1.3% compare favorably with both neoadjuvant ICI trials (grade ≥3 post-operative adverse events 18–28%) and historical chemoradiotherapy salvage series. The markedly higher pCR after ALK-TKIs likely reflects clonal homogeneity and deeper target inhibition compared with EGFR-mutant disease, where pre-existing T790M subclones can persist despite first- or second-generation TKIs. Notably, population-level data from the National Cancer Database demonstrated zero peri-operative deaths among 276 salvage lobectomies after stereotactic body radiotherapy but a higher late hazard of cardiorespiratory morbidity [28]. Our findings suggest that prior ICI/TKI exposure does not magnify the immediate surgical risk beyond accepted lobectomy benchmarks and that minimally invasive approaches—achieved in almost half of our patients—can safely be pursued despite nodal arthrofibrosis.

The oncologic relevance of the 27% pCR rate is underscored by a 2024 JNCI meta-analysis of 6530 patients in 20 neoadjuvant studies, which found the pCR to halve the risk of death (HR ≈ 0.49) and the major pathologic response (≤10% viable tumor) to confer an even stronger survival advantage [29]. Consequently, the substantial proportion of pCR observed here supports the hypothesis that resection eradicates residual resistant clones in a subset of deep systemic responders, converting cytostatic control into a durable cure.

Short-term survival after salvage surgery is encouraging: the pooled 1-year OS 88% and DFS 68% exceed historical systemic-only benchmarks and approach results from peri-operative immunotherapy trials in resectable disease (24-month EFS 62% in KEYNOTE-671; 24-month OS 73% in AEGEAN; 2-year OS 81% in NADIM II) [26,27,29]. Although cross-study comparisons must be interpreted cautiously, these parallels suggest that, in selected patients, delayed surgical consolidation can “reset the clock” and deliver survival curves akin to those attained when surgery precedes systemic therapy.

Finally, timing and patient selection emerge as critical determinants of benefit. The inverse correlation between therapy duration and pCR in our meta-regression aligns with peri-operative protocols such as AEGEAN, in which surgery is scheduled promptly after four cycles once maximal radiologic response is evident [29,30]. Early referral prevents the fibro-sclerotic fixation of hilar structures and limits the window for systemic escape. Prospective registries should therefore stratify candidates by molecular subtype, depth of response, and interval from best response to resection to delineate a therapeutic “sweet spot” where operative risk is low and incremental oncologic gain is highest. 

### 4.2. Limitations

Evidence is limited by the retrospective design, small sample sizes, and substantial heterogeneity in systemic-therapy regimens, response assessment, and surgical technique. Reporting bias favored high-volume academic centers. Few studies provided long-term follow-up beyond 36 months, precluding a robust meta-analysis of 5-year survival. Also, the absence of matched non-surgical controls limits causal inference; improved outcomes may partly reflect immunologically “hot” tumors already prone to favorable prognosis. Moreover, we excluded cohorts treated primarily with concurrent chemoradiation because post-radiation vascular and parenchymal fibrosis represents a distinct biological substrate and surgical risk profile that would have confounded the immunologic or molecular effects specifically attributable to ICIs or TKIs. Lastly, salvage candidates necessarily represent a highly selected subset with excellent performance status and robust systemic responses, likely inflating peri-operative and survival outcomes relative to the broader unresectable NSCLC population.

## 5. Conclusions

Salvage pulmonary resection after ICI or TKI conversion is a viable option for highly selected patients with initially unresectable NSCLC, achieving high R0 and pCR rates with acceptable morbidity. Multidisciplinary algorithms should integrate early surgical evaluation once systemic response plateaus or oligoprogression emerges. Future prospective registries should standardize eligibility, operative reporting, and patient-reported outcomes to delineate the true survival contribution of surgery in the era of precision oncotherapy.

## Figures and Tables

**Table 1:** Baseline and Surgical Characteristics (*n* = 312).

#	First Author (Year)	Country	Systemic Modality	*n*	Stage at Baseline	Minimally Invasive (%)	R0 Resection (%)	Pathologic CR (%)
1	Ueno 2022 [3]	JP	PD-1/PD-L1 ICI	11	IIIb–IV	27	91	18
2	Goto 2024 [22]	JP	ICI (mixed)	14	IIIb–IV	50	93	29
3	Beattie 2021 [2]	US/CH	ICI (mixed)	27	IIIb–IV	41	96	19
4	Suzuki 2023 [5]	JP	ICI ± TKI	86	III–IV	38	92	26
5	Ohtaki 2021 [6]	JP	EGFR/ALK TKI	36	IV	22	95	17
6	Takenaka 2024 [7]	JP	ICI ± TKI	18	IIIb–IV	44	94	33
7	Mangiameli 2024 [8]	IT	ICI/TKI	12	III–IV	58	92	25
8	Bott 2019 [9]	US	Nivolumab	20	IV/oligoprog.	35	100	43
9	Tong 2022 [10]	US	Pembrolizumab	25	IB–IIIA	40	96	40
10	Lücke 2020 [12]	DE	ICI (mixed)	10	III–IV	60	90	30
11	Lococo 2023 [14]	IT	Alectinib	10	IIIB–IVB	40	90	50
12	Nemeth 2024 [21]	DE/US	ICI (mixed)	24	III–IV	46	94	28
13	Sano 2023 [15]	JP	ICI (PD-1)	8	IIIb–IV	30	88	25
14	Smith 2022 [1]	UK	Pembrolizumab	9	IIIb–IV	56	89	22

ICI: immune checkpoint inhibitor; TKI: tyrosine kinase inhibitor; R0: complete (margin-negative) resection; pCR: pathologic complete response; JP: Japan; US: United States; IT: Italy; DE: Germany; UK: United Kingdom; CH: Switzerland.

**Table 2:** Peri-operative Metrics.

#	First Author (Year)	Operative Time (min, median)	Blood Loss (mL, median)	Major Complication ≥ III (%)	30-Day Mortality (%)	LOS (Days, median)
1	Ueno 2022 [3]	210	320	9	0	8
2	Goto 2024 [22]	280	400	14	0	9
3	Beattie 2021 [2]	195	290	11	0	6
4	Suzuki 2023 [5]	225	310	10	1.2	7
5	Ohtaki 2021 [6]	215	330	12	0	7
6	Takenaka 2024 [7]	200	270	8	0	6
7	Mangiameli 2024 [8]	165	180	7	0	5
8	Bott 2019 [9]	260	350	15	0	7
9	Tong 2022 [10]	180	250	9	0	5
10	Lücke 2020 [12]	170	210	10	0	4
11	Lococo 2023 [14]	205	280	13	0	6
12	Nemeth 2024 [21]	190	260	11	0	6
13	Sano 2023 [15]	220	340	10	0	8
14	Smith 2022 [1]	175	240	22	0	9

LOS: length of hospital stay; CD ≥ III: Clavien–Dindo grade III or higher complication; min: minutes; mL: milliliters.

**Table 3:** Oncologic Outcomes.

#	First Author (Year)	Nodal Down-Staging (%)	1-Year DFS (%)	1-Year OS (%)
1	Ueno 2022 [3]	45	67	85
2	Goto 2024 [22]	38	70	88
3	Beattie 2021 [2]	42	69	87
4	Suzuki 2023 [5]	40	66	86
5	Ohtaki 2021 [6]	36	62	82
6	Takenaka 2024 [7]	44	71	90
7	Mangiameli 2024 [8]	41	68	89
8	Bott 2019 [9]	55	72	92
9	Tong 2022 [10]	50	75	93
10	Lücke 2020 [12]	48	70	90
11	Lococo 2023 [14]	35	60	84
12	Nemeth 2024 [21]	39	65	87
13	Sano 2023 [15]	37	63	86
14	Smith 2022 [1]	33	61	84

DFS: disease-free survival; OS: overall survival; %: percent.

**Table 4:** Systemic Therapy.

#	First Author (Year)	Systemic Duration (mo, Median)	Cycles/Lines (Median)	Therapy Line (1st/2nd)	Best RECIST	Interval Last-Dose → Surgery (wk)	Post-op Systemic (%)
1	Ueno 2022 [3]	6.4	11	11/0	PR	6	36
2	Goto 2024 [22]	8.9	16	14/0	PR	4	42
3	Beattie 2021 [2]	7.8	13	24/3	PR	5	38
4	Suzuki 2023 [5]	10.3	14	74/12	PR	6	41
5	Ohtaki 2021 [6]	7.6	12	31/5	PR	5	43
6	Takenaka 2024 [7]	6.1	9	17/1	PR	4	39
7	Mangiameli 2024 [8]	5.7	8	11/1	PR	6	37
8	Bott 2019 [9]	8.3	11	0/20	PR	4	47
9	Tong 2022 [10]	6.2	9	25/0	PR	5	44
10	Lücke 2020 [12]	5.9	8	9/1	PR	6	49
11	Lococo 2023 [14]	7.1	12	9/1	PR	5	46
12	Nemeth 2024 [21]	9.4	17	0/24	PR	4	52
13	Sano 2023 [15]	8.2	11	7/1	PR	6	42
14	Smith 2022 [1]	7.2	12	8/1	PR	5	44

mo: months; wk: weeks; RECIST: Response Evaluation Criteria in Solid Tumors; PR: partial response; Post-op: post-operative.

**Table 5:** Pooled Operative Metrics.

Outcome	Pooled Mean or Proportion (95% CI)	Between-Study I^2^
Operative Time (min)	182 (155–211)	58%
Blood Loss (mL)	268 (190–352)	62%
Major Complication (CD ≥ III)	11% (7–16%)	21%
Prolonged Air-Leak > 5 d	9% (5–14%)	18%
Median LOS (days)	7.2 (5–10)	N/A

CI: confidence interval; CD ≥ III: Clavien–Dindo grade III or higher; LOS: length of hospital stay; N/A: not applicable.

**Table 6:** Pooled Estimates for Oncologic Outcomes.

Dataset	Endpoint	Pooled Estimate (95% CI)
**All Populations**	R0 Resection	93% (89–97%)
	Pathologic Nodal Down-Staging	41% (31–52%)
	1-Year DFS	68% (60–75%)
	1-Year OS	88% (81–93%)
	2-Year OS	76% (65–85%)
**ICI Subgroup** *(n = 222)*	R0 Resection	94% (90–97%)
	pCR	25% (18–32%)
	1-Year DFS	69% (61–76%)
	1-Year OS	89% (82–94%)
	2-Year OS	78% (66–86%)
**TKI Subgroup** *(n = 90)*	R0 Resection	91% (84–96%)
	pCR	32% (18–48%)
	1-Year DFS	65% (52–76%)
	1-Year OS	85% (75–92%)
	2-Year OS	72% (56–83%)

R0: complete (margin-negative) resection; pCR: pathologic complete response; DFS: disease-free survival; OS: overall survival; ICI: immune checkpoint inhibitor; TKI: tyrosine kinase inhibitor; %: percent; CI: confidence interval.

## Data Availability

Not applicable.
